# The limit of tolerable micromotion for implant osseointegration: a systematic review

**DOI:** 10.1038/s41598-021-90142-5

**Published:** 2021-05-24

**Authors:** Nupur Kohli, Jennifer C. Stoddart, Richard J. van Arkel

**Affiliations:** Department of Mechanical Engineering, Imperial College London, South Kensington Campus, London, SW7 2AZ UK

**Keywords:** Biological models, Bone, Preclinical research, Biomedical engineering, Implants

## Abstract

Much research effort is being invested into the development of porous biomaterials that enhance implant osseointegration. Large micromotions at the bone-implant interface impair this osseointegration process, resulting in fibrous capsule formation and implant loosening. This systematic review compiled all the in vivo evidence available to establish if there is a universal limit of tolerable micromotion for implant osseointegration. The protocol was registered with the International Prospective Register for Systematic Reviews (ID: CRD42020196686). Pubmed, Scopus and Web of Knowledge databases were searched for studies containing terms relating to micromotion and osseointegration. The mean value of micromotion for implants that osseointegrated was 32% of the mean value for those that did not (112 ± 176 µm versus 349 ± 231 µm, *p* < 0.001). However, there was a large overlap in the data ranges with no universal limit apparent. Rather, many factors were found to combine to affect the overall outcome including loading time, the type of implant and the material being used. The tables provided in this review summarise these factors and will aid investigators in identifying the most relevant micromotion values for their biomaterial and implant development research.

## Introduction

Metallic protheses implanted directly into bone have revolutionised the treatment of dental, orthopaedic and spinal disease, pain and trauma, with millions of procedures performed annually worldwide^1^. This has resulted in a continued drive from research centres of excellence and industry to develop new technology that improves outcomes, reduces revision rates, and enables treatment for more patients’ groups, such as those that are younger and more active.

In silico and in vitro modelling are at the heart of the pre-clinical development process for new implant technologies. In the field of implant fixation, a common parameter investigated is the amount of oscillatory micromotion at the bone-implant interface^2–4^. Micromotion is the temporary localised relative movement that occurs between an implant surface and adjacent bone when functional loading is applied^5,6^; with any permanent displacement known as subsidence/migration. These sub-millimetre (hence micro) motions are too small to be seen by the naked eye. Micromotion is the result of primary implant instability and differing bone/implant material moduli, and consequently depends on the implant material, bone density, implant/bone geometry, surgical technique, and the level of interference fit, as well as the magnitude and direction of the applied loading^4–9^.

Micromotion is investigated as in vivo data suggest that too much of it leads to failed implant osseointegration: a fibrous capsule forms around the implant rather than a direct structural and functional connection between the host bone and implant^10,11^. Failed osseointegration leads to aseptic implant loosening, implant failure and the need for expensive revision surgery, both financially and in terms of quality of life^12^. Thus, much research time and resource has been invested to ensure new implant designs and surgical techniques result in acceptable/improved micromotion at the bone-implant interface^13–22^. There even exists an ASTM standard (F2537–06) to ensure micromotion is measured accurately and repeatably.

Micromotion data are often compared to a limit, below which the implant is considered to pass/be suitable for clinical use, and above which it is considered to be at risk of failed osseointegration. Early in vivo research suggested an upper limit of 150 µm of micromotion and over time this value has become an oft-cited gold standard^3,23–28^. However, the evidence in the 1990s for the 150 µm limit was inconclusive and much time has subsequently elapsed meaning that there is likely much more data available with which to draw conclusions about the relationship between micromotion and osseointegration^29–31^.

This systematic review aimed to compile all the quantitative in vivo evidence relating micromotion to osseointegration to answer the following research questions: (1) Is there value of micromotion that can be universally used as a limit for in vitro or in silico modelling? (2) To what extent is micromotion correlated with bone-implant contact? (3) Which factors influence the relationship between micromotion and osseointegration?

## Results

### Study selection and characteristics

**Figure 1:**
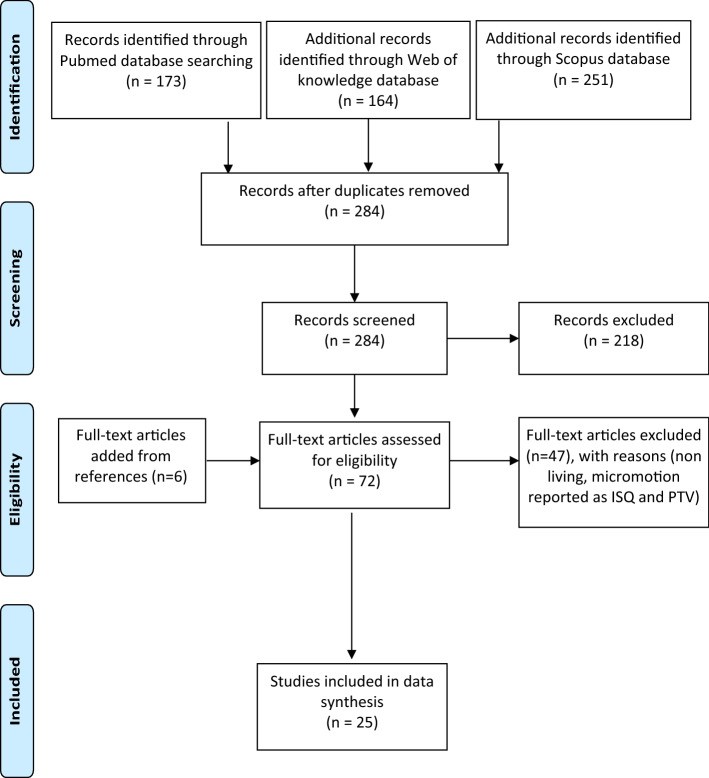
Flowchart of the study selection process.

284 unique records were identified from the databases (Fig. 1). After initial screening, 218 articles were excluded leaving 66 studies for full article screening. An additional 6 studies that passed all inclusion criteria were identified from these 66 studies. After full-text screening, 25 studies were found to be eligible for the quantitative analysis (Table 1).

**Table 1:** Osseointegrated (OI) and non-osseointegrated (Non-OI) values of micromotion (µm) from the studies selected

Author	Year	Country	Micromotion OI (µm)	Micromotion Non-OI (µm)	Applied or measured	Animal or Human
Aspenberg^32^	1992	Sweden	N/A	500	Applied	Animal
Bragdon^33^	1996	USA	20	40,150		
Duyck^34^	2006	Belgium	60	30,90		
Goodman a^35^	1995	USA	N/A	500,500		
Goodman b^31^	1993	Sweden	750	750		
Goodman c^36^	1994	Sweden	500*0	500		
Goodman d^37^	1993	Sweden	500	500		
Jakobsen a ^38^	2015	Denmark	N/A	500		
Jakobsen b^39^	2017	Denmark	N/A	500,500		
Jasty^40^	1997	USA	20	40,150		
Kawahara^29^	2003	Japan	30	580,630		
Overgaard^41^	1996	Denmark	150*0	150 *12		
Soballe a^42^	1992	Denmark	N/A	500,500		
Soballe b^43^	1992	Denmark	150	150		
Vandamme a^30^	2007	Belgium	30,50	30		
Vandamme b^44^	2007	Belgium	30,90	N/A		
Vandamme c^45^	2008	Belgium	30,30	N/A		
Vandamme d^46^	2007	Belgium	30,30	N/A		
Manley^25^	1995	USA	33 ± 23.7, 17 ± 4.2	N/A	Measured	
Pilliar^47^	1986	Canada	28	150		
Trisi a^48^	2017	Italy	77.9 ± 17.29, 75.3 ± 19	N/A		
Trisi b^8^	2015	Italy	64 ± 27	177 ± 87		
			15 ± 5 ,22 ± 6	N/A		
Trisi c^49^	2016	Italy	94.88 ± 10.94, 60.45 ± 5.29	N/A		
Trisi d^50^	2016	Italy	161.26 ± 134.39	619.5 ± 328.26		
Engh^51^	1992	USA	< 40	150	Measured	Human

For applied values, the value was set as a controlled experimental parameter, for measured values means and standard deviation are reported where possible. *0 represents experiments with immobilized implants after a period of loading. *12 represents experiments that applied an additional implant displacement for 12 weeks.

### Micromotion and osseointegration

**Figure 2:**
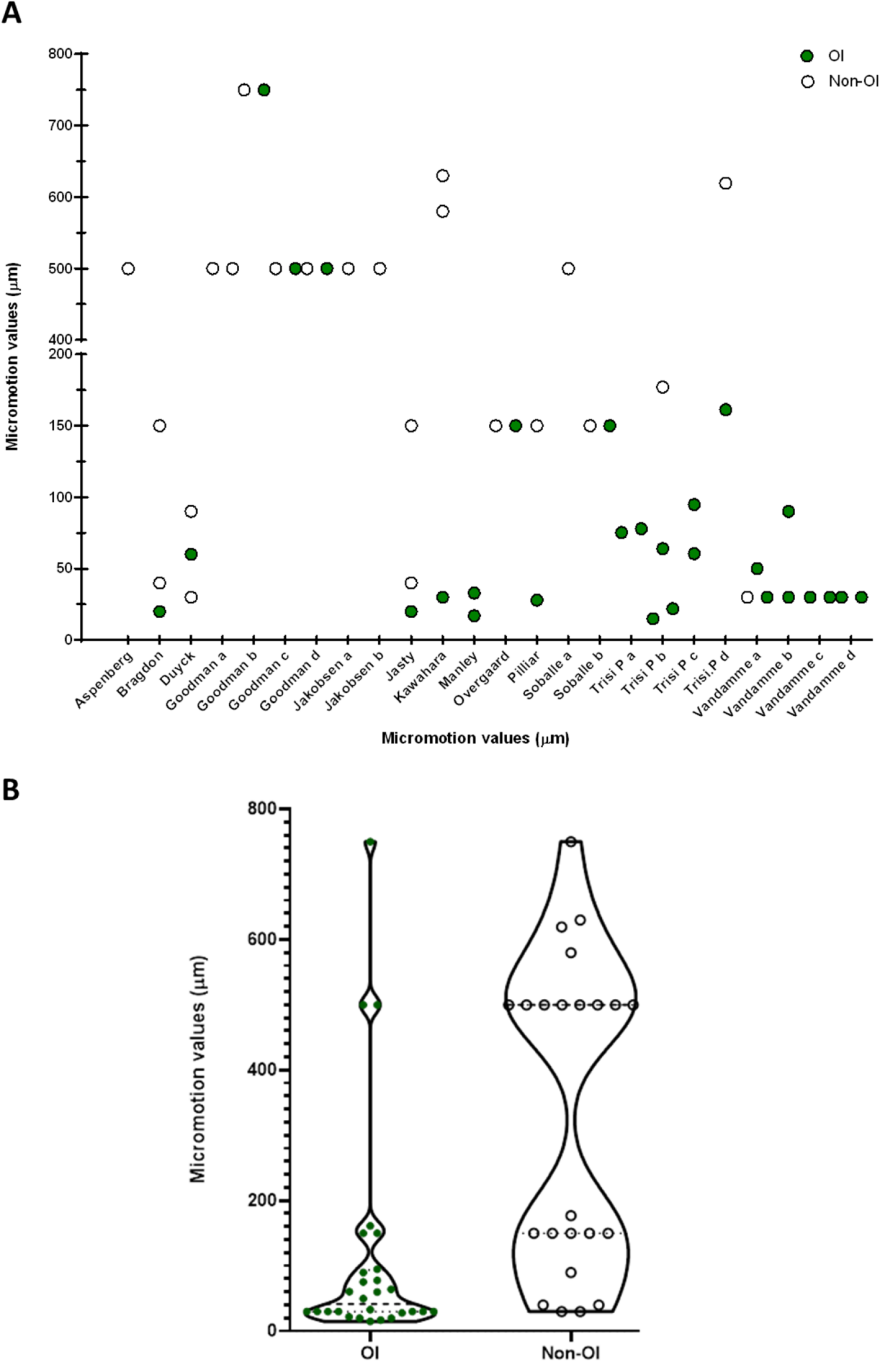
(**A**) Scatterplot of the animal data showing the micromotion value for osseointegrated (green, circle, n = 28) and non-osseointegrated (hollow circles, n = 23) samples. (**B**) Violin plot of the same data. Whilst micromotion was lower for the osseointegrated samples (Mann Whitney test *p* < 0.001), there was also considerable overlap between the groups.

One human and twenty-four animal studies were identified (Table 1). For the human post-mortem study, the micromotion for osseointegrated hip stems was less than 40 µm which compared to 150 µm for a stem with failed bone ingrowth. For the animal studies, the mean value of micromotion for implants that osseointegrated was 32% of the mean value for those that did not (112 ± 176 µm osseointegrated versus 349 ± 231 µm non-osseointegrated, Mann Whitney test *p* < 0.001, Fig. 2). However, the osseointegration outcome also depended on other experimental/implant conditions with no distinct osseointegration limit detected. Rather, the range for successful/failed osseointegration overlapped: 15 to 750 µm for osseointegrated samples versus 30 to 750 µm for non-osseointegrated samples (Table 1 and Fig. 2).

### The effects of research method: applied vs measured micromotion

**Figure 3:**
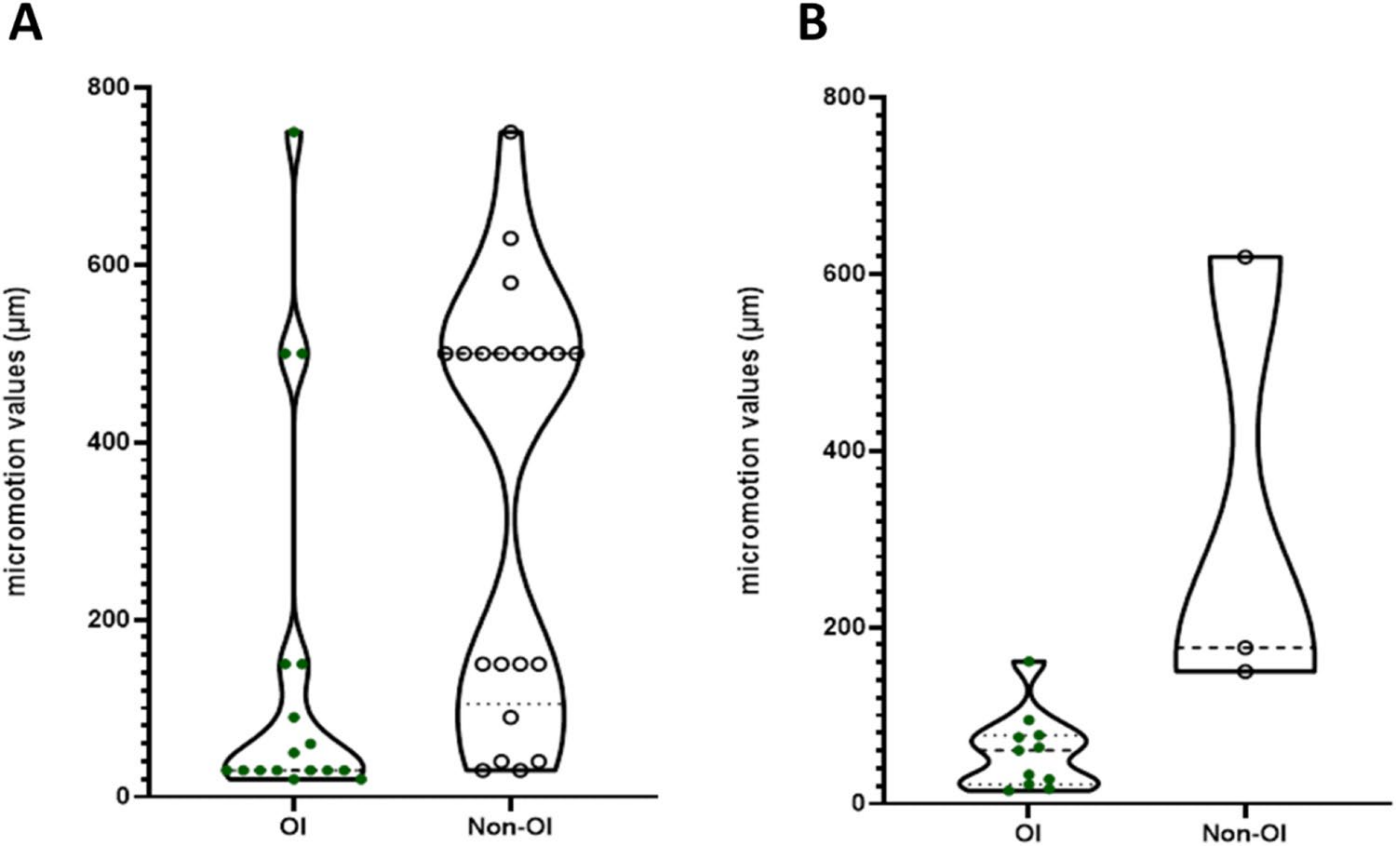
(**A**) Applied values of micromotion in osseointegrated (OI, n = 17) and non-osseointegrated (Non-OI, n = 20) groups for the animal studies. Mann Whitney *p* value = 0.001 **. (**B**) Measured values of micromotion in OI (n = 11) and non-OI (n = 3) for the animal studies. Mann Whitney *p* value = 0.003 **.

When micromotion was applied, lower micromotion resulted in more consistent osseointegration (Fig. 3A, Mann Whitney *p* value = 0.001). Similarly, when micromotion was measured at the end of the study duration, implants that osseointegrated had lower micromotion than implants that did not (Fig. 3B, Mann Whitney *p* value = 0.01). Comparing values of micromotion between the methods (measured vs applied), no differences were observed for the osseointegrated group, and similarly there was no difference between the methods for the non-osseointegrated group. (Fig. 3).

### Micromotion and bone-implant-contact

Out of the 24 animal studies, 13 studies examined osseointegration as the percentage of BIC, 2 studies reported on bone ingrowth and 1 study reported on both BIC and bone ingrowth. For implants that were defined as osseointegrated, a positive correlation was observed between micromotion and % BIC (Spearman’s *ρ* = 0.41, *p* value = 0.02). Micromotion and BIC were not correlated for the non-osseointegrated group (*p* value = 0.39), nor the full dataset (*p* value = 0.07).

### Observation time and Bone-implant-contact

There was a positive correlation between observation time and percentage BIC, with longer study duration time resulting in better percentage BIC (Spearman’s *ρ* = 0.40, *p* value = 0.01).

## Discussion

The most important finding of this systematic review was that the available data refutes the idea of a universal limit of tolerable micromotion for implant osseointegration. Whilst on average, the micromotion associated with osseointegration was 32% of the micromotion associated with failed fixation, many exceptions to the rule were identified (Fig. 2). In some studies, micromotion at the bone-implant interface as high as 750 µm osseointegrated, whilst in other micromotion as low as 30 µm did not osseointegrate. Thus, implant and external factors must be considered when estimating the level of micromotion that could lead to successful osseointegration for a new biomaterial/implant. The following implant factors were associated with higher levels of micromotion and successful osseointegration: hydroxyapatite coating^43^, larger threads in lower density bone^8^, and square pore cross-sectional shape^37^. The following environmental factors were associated with higher levels of micromotion and successful osseointegration: infrequent loading^31^, a rest period following initial loading^41^ and longer study duration (9 weeks or more)^30,44^ (Table 2).

**Table 2:** Detailed study characteristics of the selected studies.

Author	Year	No. of animals or patients	Species	No. of samples (per group)	No. of study groups	Implant material	Implant coating or implant type	Bone	Time (weeks)	Loading conditions	Loading cycles and time	Micromotion (µm)		Bone ingrowth	Bone-implant-contact
												Osseo-integrated	Non-osseo-integrated		
Aspenberg	1992	6	rabbits	13	2	titanium	none	Tibia	3	Unloaded	N/A	Unloaded			Not measured
				15					3	500 µm Micromotion	20 cycles/day		500		
Bragdon	1996	20	Dogs	5	4	Titanium	None	Femur	6	UNLOADED	N/A	Unloaded			Not measured
									6	20 µm Micromotion	8 h/day	20			
									6	40 µm micromotion	8 h/day		40		
									6	150 µm micromotion	8 h/day		150		
Duyck	2006	10	Rabbits	10	4	Titanium	None	Tibia	6	Unloaded		Unloaded			20–25%
									6	30 µm micromotion	800 cycles/day; twice/week		30		5–10%
									6	60 µm micromotion	800 cycles/day; twice/week	60			15–20%
									6	90 µm micromotion	800 cycles/day; twice/week		90		5–10%
Goodman a	1995	9	Rabbits	9	4	Titanium	Micromotion alone	Femur	3	500 µm micromotion	40 cycles/day		500	25 ± 6	
							Polyethylene particles only		3	Unloaded	N/A		Unloaded	23 ± 9	
							No polyethylene		3	Unloaded	N/A	Unloaded		33 ± 6	
							polyethylene + micromotion		3	500 µm micromotion	40 cycles/day		500	23 ± 9	
Goodman b	1993	7	Rabbits	7	3	Titanium	None		3	Unloaded		Unloaded			31 ± 2%
				10				Tibia	3	750 µm micromotion	20 cycles/day	750			46 ± 5%
				7					3	750 µm micromotion	20 cycles twice/day		750		19 ± 7%
Goodman c	1994	5	Rabbits	5	3	Titanium	None	Tibia	6	500 µm micromotion (3 weeks), then unloaded (3 weeks)	40 cycles/day then unloaded	500			37 ± 7
									3	500 µm micromotion			500		20 ± 2
									3	Unloaded	N/A	unloaded			37 ± 6
Goodman d	1993	10	Rabbits	6	2	Titanium	Square chamber	Tibia	3	500 µm micromotion	20 cycles/day	500			not measured
				5			Round chamber		3	500 µm micromotion	20 cycles/day		500		not measured
Jakobsen a	2015	10	Sheep	10	2	PMMA		Femur	12	500 µm micromotion	Every gait cycle	Unloaded	500		
Jakobsen b	2017	10	Sheep	10	2	PMMA	Control	Femur	12	500 µm micromotion	Every gait cycle		500		
							Zoledronate		12	500 µm micromotion	Every gait cycle		500		
Jasty	1997	20	Dogs	5	4	Titanium	None	Femur	6	Unloaded	N/A	Unloaded		9.3 ± 2.3	
									6	20 µm micromotion	8 h/day	20		9.0 ± 3.1	
									6	40 µm micromotion	8 h/day		40	11.8 ± 3.9	
									6	150 µm micromotion	8 h/day		150	10.4 ± 3.0	
Kawahara	2003		Beagles			Titanium	None	Mandi-ble	6	8 N	10 s	30	580, 630		not measured
Overgaard	1996	14	Dogs	7	2	Titanium	Hydroxyapatite coated	Femur	16	150 µm micromotion (4 weeks), then unloaded (12 weeks)	Everyday	150		28.5 ± 8.8	54.6 ± 10.0
									16	150 µm micromotion	Everyday		150	24.1 ± 16.1	37.7 ± 10.1
Soballe	1992	14	Dogs	8	4	Titanium	Hydroxyapatite coated	Femur	4	500 µm micromotion	every gait cycle		500		0–10%
							Hydroxyapatite coated		4	Unloaded	N/A	unloaded			45%
							Titanium coated		4	500 µm micromotion	every gait cycle		500		0–10%
							Titanium coated		4	Unloaded	N/A	Unloaded			0–10%
Soballe	1992	14	Dogs	7	4	Titanium	Hydroxyapatite coated	Femur	4	150 µm micromotion	Every gait cycle	150			7 ± 2
							Hydroxyapatite coated		4	unloaded	N/A	unloaded			65 ± 2
							Titanium coated		4	150 µm micromotion	Every gait cycle		150		0
Vandamme a	2007	14	Rabbits	10	3	Titanium	None	Tibia	12	Unloaded	N/A		unloaded		0–20%
				10					6	30 µm micromotion	400 cycles/day ; twice/week		30		0–20%
				11					12	30 µm micromotion (6 weeks), then 50 µm micromotion (6 weeks)	400 cycles/day; twice/week, then 800 cycles/day; twice/week	30, 50			60–80%
Vandamme b	2007	10	rabbits	10	3	Titanium	none	Tibia	9	Unloaded		unloaded			42.22
									9	30 µm micromotion	400 cycles/day; thrice/week	30			71.43
									9	90 µm micromotion	400 cycles/day; thrice/week	90			74.36
Vandamme c	2008	20	Rabbits	10	2	Titanium	Turned	Tibia	9	Unloaded	N/A	unloaded			6.98
							Turned		9	30 µm micromotion	400 cycles/day; thrice/week	30			53.33
							Roughened		9	Unloaded	N/A	Unloaded			42.22
							Roughened		9	30 µm micromotion	400 cycles/day; thrice/week	30			71.43
Vandamme d	2007	10	Rabbits	10	3	Titanium	screw	Tibia	9	Unloaded	N/A		unloaded		0–3%
							screw		9	30 µm micromotion	400 cycles/day; thrice/week	30			9–20%
							cylindrical		9	30 µm micromotion	400 cycles/day; thrice/week	30			0–8%
							titanium		4	Unloaded	N/A	Unloaded			13 ± 3
Manley	1995	12	Dogs	6	2	Titanium	Collared	Femur	16	± 50 N	16 s at 0.5 Hz	33 ± 23.7			52 ± 11.4
							Collarless		16	± 50 N	16 s at 0.5 Hz	17 ± 4.2			42 ± 8.5
Pilliar	1986		Dogs	5	3	Cobalt Chrome		Femur	52			28	150		
Trisi a	2017	2	sheep	10	2	Titanium	SLA	Iliac crest	8	25 N/mm	End point analysis	77.9 ± 17.29			49.49 ± 7.70
							FEL		8	25 N/mm	End point analysis	75.3 ± 19			65.33 ± 6.35
Trisi b	2015	4	Sheep	20	2	Titanium	Large threaded	Iliac crest	8	25 N/mm	End point analysis	64 ± 27			50.58 ± 8.65
							small threaded		8	25 N/mm	End point analysis		177 ± 87		40.98 ± 14.03
							Large threaded	Mandi-ble	8	25 N/mm	End point analysis	15 ± 5			36.1 ± 18.3
							small threaded		8	25 N/mm	End point analysis	22 ± 6			34.06 ± 18.18
Trisi c	2016	2	Sheep	10	2	Titanium	Coventional drill	Iliac crest	8	25 N/mm	End point analysis	94.88 ± 10.94			46.19 ± 3.98
							Osseo-densification		8	25 N/mm	End point analysis	60.45 ± 5.29			49.58 ± 3.19
Trisi d	2016	2	Sheep	24	2	Titanium	Healthy	Iliac crest	8	25 N/cm	End point analysis	161.26 ± 134.39			44.75 ± 9.77
							Failed		8	25 N/cm	End point analysis		619.5 ± 328.26		22.6 ± 9.54
Engh	1992	14 (6 female)	Human, mean age 71	14	1	Cobalt Chrome	Coated hip stem	Femur	52–403	Gait & stair climbing	N/A	< 40	150		

The gold standard micromotion limit is often considered 150 µm. However, Overgaard et al., and Soballe et al., showed that this level of micromotion can be tolerated if a period of rest is allowed after initial loading and if the implant was coated with hydroxyapatite^41,43^. The accelerated resorption of HA coating under excessive micromotion could have led to a better bony ingrowth as studies have previously shown that HA coating on titanium implants improve BIC through its direct interaction with osteoblast, osteoclasts and pro-inflammatory markers^52,53^. Further, previous studies have also shown that a period of rest or otherwise referred to as recovery phase may be beneficial for BIC. The recovery phase or time off helps to counteract the waning effects of long-term mechanical loading, and improve the responsiveness of osteoblasts and osteocytes to restart bone formation^54,55^. Goodman et al., showed that oscillatory motions up to 750 µm once a day would allow successful osseointegration, while the same motions twice a day would not^31^, emphasizing the effect of loading duration. Similarly, Goodman also demonstrated how implant factors can influence the tolerable micromotion: by changing the pore-cross sectional shape of the outer bone chamber from round to square in the traditional bone chamber designs, bony ingrowth would be facilitated even with micromotions as high as 500 µm.

**Figure 4:**
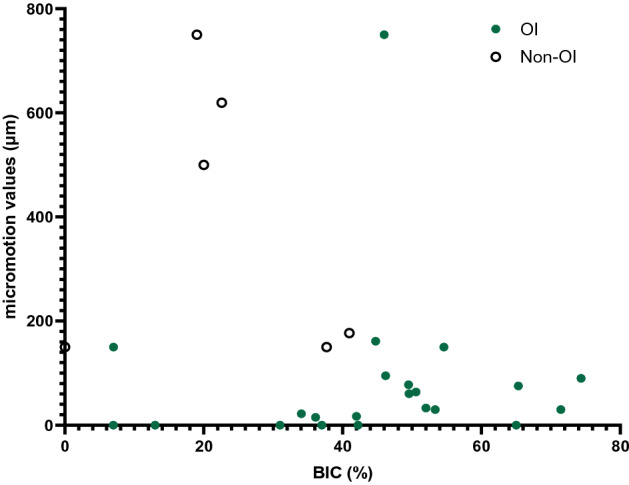
Scatter plot graph of the correlation between micromotion values and percentage BIC for the animal studies. For the osseointegrated data (green filled circles) a positive correlation was found (Spearman’s *ρ* = 0.41, *p* value = 0.02). No correlation was observed for the non-osseointegrated data (empty circles), nor the full dataset (all circles).

Whilst the data reviewed revealed that the mean value of micromotion for successful osseointegration was 112 µm, some studies showed that micromotion as low as 30 µm can lead to failed implant fixation^30,40^. The authors attributed this to the duration of loading, hypothesizing that the process of bone formation had not been reached within 6 weeks. Subsequent experiments measured osseointegration at 9 weeks or more and demonstrated successful osseointegration with the longer study duration^30,44^. The data from this systematic review further supports this finding, demonstrating a positive correlation between osseointegration (measured by percentage BIC) and study duration (Fig. 4). Biologically, osseointegration starts with woven bone formation, followed a period of remodelling to lamellar bone in response to mechanical loading. This transition from woven bone to lamellar bone formation takes starts around 6–8 weeks and can take a period of months to complete^56,57^. Therefore, in vivo experimental studies exploring osseointegration of implants should allow a time period of over 6 weeks to see the full healing response. The effects of study duration have also been reflected by recent computational research which highlighted the differing mechanisms between bone healing and remodelling, and hence the importance of the measurement time point^2^.

**Figure 5:**
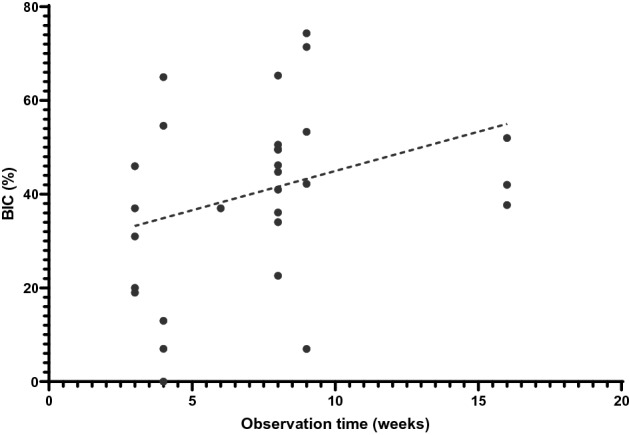
Scatter plot demonstrating the correlation between observation time and percentage BIC for the animal studies. Spearman’s *ρ* = 0.40, *p* value = 0.01.

Another explanation for the contradictory in vivo data is that micromotion is a simplified, clinically convenient measure, which overlooks the fundamental mechanobiological mechanisms that drive implant osseointegration. In vivo data coupled with finite element analyses suggest that it is the interfacial stress–strain state resulting from implant micromotion that stimulates osseointegration^58,59^. Different loading conditions (axial, shear, torsional, etc.), combined with different localised implant/bone geometry lead to different stress–strain states, with too much strain leading to fibrous tissue formation^58–61^. Indeed, by considering the interfacial stress–strain state, it is possible to relate implant bony ingrowth theory^2,58,59^ to fracture healing theory^62–65^, which intuitively one would expect given the involvement of the same cell types^66^. Conversely, when the implant/environmental conditions that affect the interfacial stress–strain state are ignored, counter-intuitive trends can be observed. For example, when neglecting these factors, it was found that increased micromotion was positively correlated with increased percentage BIC (Fig. 5). However, within study data demonstrate that micromotion and percentage BIC are negatively correlated^50^. This further emphasises the needs to consider implant and environmental factors and their link to the interfacial stress–strain state when interpreting how micromotion affects osseointegration. It should also be noted that there is no standard interpretation of BIC and so caution should also be applied when interpreting BIC between studies. Some studies report BIC as the fraction of mineralized bone in direct contact with the implant surface^44^, whilst other describe it as the length of the implant surface in contact with (both mineralised and non-mineralised) bone relative to the total implant length^34^.

Historically, the causal effect of implant micromotion on osseointegration was investigated by applying known displacement and subsequently measuring osseointegration. However, more recently, research method has shifted to applying known loads and quantifying micromotion at the end of the experiment^4,8^. Both measurement techniques were able to identify differences in micromotion between implants that osseointegrated and those that did not. Interestingly, when comparing the results between the two different methods, no differences were found.

The majority of studies identified applied micromotion as a controlled experimental condition, meaning that mean and standard deviation micromotion data were not available prohibiting application of the established meta-analysis approaches recommended by Borenstein *et al*^67^. For the same reason, it was not possible to perform a cumulative meta-analysis to quantify the risk of bias between studies. Rather, to provide some quantitative analysis we applied Mann Whitney tests to the data extracted from each study. Further we isolated the effects of studies that applied micromotion, and those that measured it, and found that this did not affect the principal finding our systematic review (Fig. 3).

In conclusion, this systematic review has demonstrated that the idea of a universal limit of tolerable micromotion for implant osseointegration is misleading. Rather, implant and environmental factors, and their link to interfacial stress–strain states, must be considered to identify the most appropriate limit for the biomaterial/patient group under consideration. The tables provided in this systematic review summarise the implant and environmental conditions for all published quantitative in vivo micromotion research and will enable investigators to compare their data to the most appropriate values.

## Materials and methods

### Protocol and registration

Prior to the investigation, the protocol was registered with the International prospective register for systematic reviews PROSPERO (ID: CRD42020196686), following the Preferred Reporting Items for Systematic Reviews and Meta-Analyses (PRISMA) statement and checklist^68^.

### Eligibility criteria

Studies which fulfilled the following criteria were included: (1) in vivo animal research or post-mortem human data where the implant was inserted pre-mortem. (2) testing of osseointegration when micromotion was either applied or measured with micromotion values reported in the form of displacement (3) the study was an original research article; and (4) the studies were published in English.

Articles were excluded if: (1) micromotion was measured indirectly and/or reported as implant stability quotient (ISQ) or resonance frequency analysis (RFA); (2) Study duration less than 3 weeks; (3) Finite element analysis (FEA) or computational studies; (4) cemented implants; (5) cadaveric bone in vitro experiments where prostheses were inserted post-mortem; and (6) synthetic bone in vitro experiments.

### Information sources and search strategy

An electronic search was performed for articles published up to 16^th^ November 2020, in the following databases: PubMed, Scopus and Web of Science. The search strategy identified papers which included the following terms: (micromotion OR "micro-motion" OR "micro motion") AND ("osseointegration" OR "osteointegration").

### Study selection

Two independent reviewers (N.K., J.S.) assessed the titles and abstracts of all the studies and discarded studies that met any of the exclusion criteria. The full text of all remaining studies was then assessed against the inclusion and exclusion criteria. Any disagreement regarding eligibility of articles were resolved by a third reviewer (R.v.A.).

### Data collection process and data items

Data relating to osseointegrated and non-osseointegrated values of micromotion were extracted. The country, animal species, number of study groups, duration of the experiment, implant material and loading conditions were recorded. The outcome of osseointegration measured as bony ingrowth or percentage bone-implant-contact (BIC) were also recorded.

The micromotion methodology (applied or measured) was also recorded. In the applied group, known values of micromotion in the form of cyclic loading were directly applied as a controlled experimental condition and then osseointegration was assessed. In measured group, micromotion was not a controlled experimental condition, rather micromotion at the bone-implant interface was measured once the implant had osseointegrated/not.

### Statistical analysis

Data were analysed and plotted using Graph Pad Prism 8 software and have been reported as mean ± standard deviation (SD). Four analyses were performed:

1. All micromotion values were grouped into osseointegrated/not, according to the definition used by the original study authors. Data were first tested for normality, and then non-parametric Mann Whitney tests were used to compare differences between groups.
2. Micromotion values were further discretised according to the study method (applied vs measured micromotion). Then analysis 1) was repeated for both of these subgroups.
3. Spearman correlation tests were used to examine correlation between percentage BIC and micromotion values for three groups: all data, osseointegrated, non-osseointegrated.
4. Spearman correlation tests were used to examine the correlation between percentage BIC and study duration.

The significance level was set to *α* = 0.05.

## Data Availability

Data generated and analysed during this study are included in this published article. Data are available from the corresponding author subject to reasonable request.
